# Postmitotic neurons develop a p21-dependent senescence-like phenotype driven by a DNA damage response

**DOI:** 10.1111/j.1474-9726.2012.00870.x

**Published:** 2012-12

**Authors:** Diana Jurk, Chunfang Wang, Satomi Miwa, Mandy Maddick, Viktor Korolchuk, Avgi Tsolou, Efstathios S Gonos, Christopher Thrasivoulou, M Jill Saffrey, Kerry Cameron, Thomas von Zglinicki

**Affiliations:** 1 Institute for Ageing and Health, Newcastle UniversityNewcastle upon Tyne NE4 5PL, UK; 2 Department of Life Sciences, The Open UniversityMilton Keynes MK7 6AA, UK; 3 National Hellenic Research Foundation, Institute of Biological Research and BiotechnologyAthens, Greece; 4 Department of Anatomy and Developmental Biology, University College LondonLondon WC1E 6DE, UK

**Keywords:** aging, brain, inflammation, oxidative stress, neurons, senescence

## Abstract

In senescent cells, a DNA damage response drives not only irreversible loss of replicative capacity but also production and secretion of reactive oxygen species (ROS) and bioactive peptides including pro-inflammatory cytokines. This makes senescent cells a potential cause of tissue functional decline in aging. To our knowledge, we show here for the first time evidence suggesting that DNA damage induces a senescence-like state in mature postmitotic neurons *in vivo*. About 40–80% of Purkinje neurons and 20–40% of cortical, hippocampal and peripheral neurons in the myenteric plexus from old C57Bl/6 mice showed severe DNA damage, activated p38MAPkinase, high ROS production and oxidative damage, interleukin IL-6 production, heterochromatinization and senescence-associated β-galactosidase activity. Frequencies of these senescence-like neurons increased with age. Short-term caloric restriction tended to decrease frequencies of positive cells. The phenotype was aggravated in brains of late-generation TERC−/− mice with dysfunctional telomeres. It was fully rescued by loss of p21(CDKN1A) function in late-generation TERC−/−CDKN1A−/− mice, indicating p21 as the necessary signal transducer between DNA damage response and senescence-like phenotype in neurons, as in senescing fibroblasts and other proliferation-competent cells. We conclude that a senescence-like phenotype is possibly not restricted to proliferation-competent cells. Rather, dysfunctional telomeres and/or accumulated DNA damage can induce a DNA damage response leading to a phenotype in postmitotic neurons that resembles cell senescence in multiple features. Senescence-like neurons might be a source of oxidative and inflammatory stress and a contributor to brain aging.

## Introduction

DNA double-strand breaks are potent inducers of a DNA damage response (DDR), characterized by activation of sensor kinases, including ataxia telangiectasia mutated (ATM) and ataxia telangiectasia and rad3 related (ATR), formation of DNA damage foci incorporating the activated histone variant H2A.X (γH2A.X), 53BP1 and others, and activation of the cell cycle checkpoint protein p53 (Shilo, 2003). In fibroblasts and other proliferation-competent cells, a persistent DDR causes an irreversible proliferation arrest termed cellular senescence (Hayflick, 1965). Importantly, senescent cells develop a phenotype that goes far beyond simple growth inhibition. Major changes in morphology, activation of the lysosomal hydrolase β-galactosidase (sen-β-Gal) and major shifts in gene expression patterns towards a pro-inflammatory secretion pattern occur in senescent cells. Moreover, mitochondrial dysfunction is induced as a necessary component of senescence leading to a multifold increase in mitochondrial ROS production, which in turn increases DNA damage levels and stabilizes the DDR in senescent cells (Passos *et al*., 2010). Thus, senescent cells are both pro-oxidant and pro-inflammatory and might thus be a potential cause of aging-related loss of function (Freund *et al*., 2010).

A complex network of signalling pathways interconnects the DDR with downstream phenotypic changes in senescence. One major signalling pathway towards mitochondrial dysfunction involves the inhibitor of cyclin-dependent kinases p21 (CDKN1A), which is activated by the DDR and triggers via GADD45 the induction of p38MAPK and TGFβ. This pathway accounts largely for the senescence-associated ROS production in human and mice fibroblasts, enterocytes and other cell types (Passos *et al*., 2010). Intact p53 and pRb tumour suppressor pathways were found to be necessary for the induction of mitochondrial dysfunction and ROS production in oncogene-induced senescence (Moiseeva *et al*., 2009), but functionally active p53 or p16 are not required for induction of the senescence-associated secretory phenotype in human and mice fibroblasts. In fact, the secretory phenotype was aggravated if p53 function was compromised (Rodier *et al*., 2009). There are contradictory data as to whether p21(CDKN1A) is necessary for induction of the senescence-associated secretory phenotype (Coppe *et al*., 2011; Rovillain *et al*., 2011). It has been shown that the pro-inflammatory secretory phenotype is driven by p38MAPK with a significant involvement of NF-κB (Freund *et al*., 2011). Both p21 (CDKN1A)-dependent and –independent activation of p38MAPK may occur in senescence. Sen-β-Gal activation appears to be downstream of the proliferation arrest, but the signalling pathway(s) involved are not known. Large-scale heterochromatinization and formation of so-called senescence-associated heterochromatin foci (SAHF) are another late consequence of a persistent DDR and senescent growth arrest (Narita *et al*., 2006; Kreiling *et al*., 2011). Together, these changes may be summarized as the senescent phenotype.

It is not known whether persistent DNA damage can induce a similar, senescence-like phenotype in postmitotic cells, for example mature neurons. Neurons both in the aging brain and in the periphery accumulate various forms of DNA damage with age (Rao, 2009). This includes the formation of DNA double-strand breaks and DNA damage foci (Sedelnikova *et al*., 2004). Reactive oxygen species levels are high in neurons because of their high metabolic activity and relatively poor antioxidant defence (Sohal & Weindruch, 1996; Finkel & Holbrock, 2000), and high ROS levels have long been implicated as a DNA-damaging agent in nonpathological brain aging (Poon *et al*., 2004) as well as in the pathogenesis of age-related neurological disorders (Schapira, 1998). We show here that in neurons from aging mice DNA damage is not simply the consequence of high oxidative damage. Rather, the DNA damage response is interconnected with multiple markers of the senescent phenotype, including ROS production, interleukin secretion, sen-β-Gal activity and heterochromatinization. As in ‘classical’ senescence, p21(CDKN1A) is a major mediator of these downstream effects. These data indicate that mature neurons develop a senescence-like phenotype with aging.

## Results

**Fig. 1:**
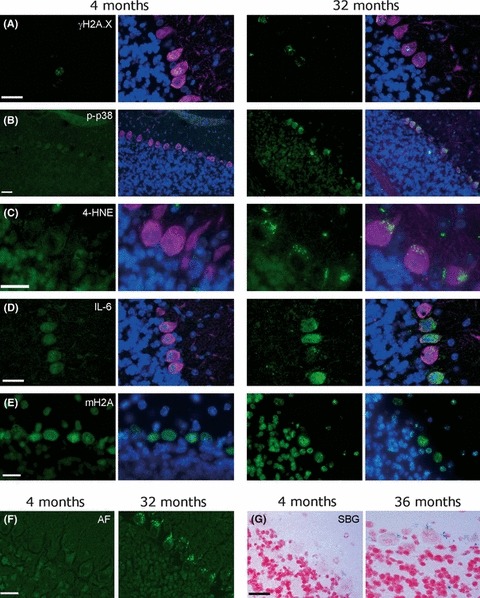
Purkinje neurons in old (32 months) but not in young (4 months) mice are positive for multiple markers of the senescent phenotype. Representative images are shown. Size marker bars indicate 20 μm. (A–E) Cerebellar sections of mice at the indicated ages were stained with DAPI (blue, nuclei), the neuronal marker calbindin (purple, omitted in E for clarity) and the antibody of interest visualized by IgG-FITC (green). Shown are the antibody of interest (left) and the merged image (right). (A) γH2A.X, (B) activated p38MAPK, (C) 4-hydroxynonenal (4-HNE, a marker for lipid peroxidation), (D) IL-6, (E) mH2A. (F) Autofluorescence on unstained sections. (G) sen-β-Gal activity. Positive cells show blue cytoplasmic staining.

Purkinje cells are the sole output neurons in the cerebellar cortex and are known to be sensitive to age-related damage (Zhang *et al*., 2010). We identified Purkinje cells by their specific location next to the granular layer in the cerebellum, their large size, low nuclear DNA density and positivity for calbindin, a common neuronal marker (Fig. 1). We confirmed frequent DNA damage in Purkinje cells from old (32 months of age), but not from young (4 months of age), mice by immunofluorescence visualization of γH2AX-containing DNA damage foci (Fig. 1A). Immunohistochemical staining with antibodies against 53BP1 confirmed the presence of a DDR in Purkinje and cortical neurons from old mice (Fig. S1). To find whether DDR induced a senescence-like phenotype in Purkinje neurons *in vivo*, we stained cerebellar sections for activated p38MAPK (Freund *et al*., 2011), 4-HNE as a marker for lipid peroxidation caused by oxidative stress, IL-6 as the most robustly upregulated pro-inflammatory cytokine in mouse cells (Coppe *et al*., 2010), the heterochromatin-associated histone mH2A (Kreiling *et al*., 2011) and sen-β-Gal as a widely accepted general marker of the senescent phenotype. In addition, we used broadband autofluorescence intensity as an independent marker of oxidative damage leading to accumulation of lipofuscin and other cross-linked material in brain (Passos *et al*., 2010). Many Purkinje neurons in old mice brains showed high levels of phospho-p38 (Fig. 1B), cytoplasmic granular accumulation of 4-HNE (Fig. 1C) and increased levels of IL-6 (Fig. 1D). Average nuclear intensity of the anti-mH2A signal was not enhanced in old brain Purkinje cells; however, granularity was enhanced and signals became concentrated in multiple nuclear foci (Fig. 1E). Whether these foci represent SAHFs is not completely clear because a Wnt-signalling pathway that regulates SAHF formation in human cells has been shown to be absent in mouse fibroblasts (Kennedy *et al*., 2010). However, accumulation of mH2A at pericentromeric heterochromatin has been shown in multiple tissues from aging mice (Kreiling *et al*., 2011). Bright autofluorescent granules, presumably secondary lysosomes, were evident in unstained sections (Fig. 1F), and there was increased activity of sen-β-Gal in Purkinje cells from old brains (Fig. 1G). None of these signals was observed by staining with isotype control antibodies or with an unrelated antibody (Fig. S2). Immunofluorescent results were confirmed by immunohistochemistry (Fig. S3).

**Fig. 2:**
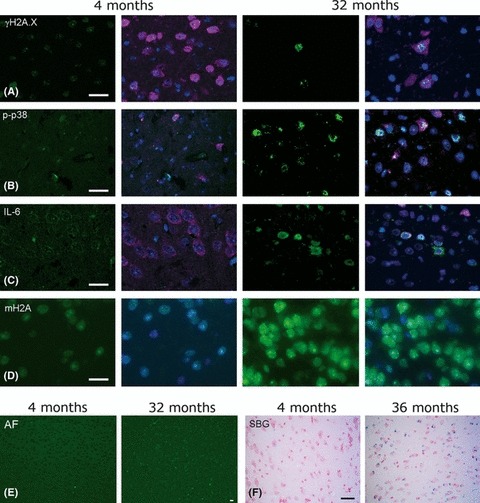
Cortical neurons in old mice are positive for multiple markers of the senescent phenotype. Representative images from 4 to 32 months old mice are shown. The size marker bars indicate 20 μm. (A–D) Cortical sections were stained with DAPI (blue), the neuronal marker calbindin (purple, omitted in D for clarity) and the antibody of interest (green). Shown are the antibody of interest (left) and the merged image (right). (A) γH2A.X, (B) activated p38MAPK, (C) IL-6, (D) mH2A. (E) Autofluorescence on unstained sections. (F) Sen-β-Gal activity (blue).

Analysis of cortical brain sections revealed a very similar picture (Fig. 2). Cortical neurons in old (32 months) mice, identified by their positivity for calbindin as well as their size and shape, were frequently positive for γH2AX foci (Fig. 2A), showed activated p38MAPK (Fig. 2B), were positive for IL-6 (Fig. 2C), showed strong focal staining for the heterochromatin marker mH2A associated with DAPI heterochromatic foci (Fig. 2D), were autofluorescent (Fig. 2E) and positive for sen-β-Gal (Fig. 2F). No positive signals were seen in cortical neurons if stained with isotype control or unrelated antibody (Fig. S2). Results were confirmed by immunohistochemistry (Fig. S4). All these markers were much reduced in cortical neurons from young mice (Fig. 2A–F). Furthermore, many neurons in the hippocampus from old mice stained positive for the same markers of a senescent phenotype (Fig. S5). However, hippocampal neurons in young mice brains also showed frequent DNA damage and heterochromatin foci, which often colocalized (data not shown). Therefore, a full quantitative analysis was not carried out in the hippocampus.

**Fig. 3:**
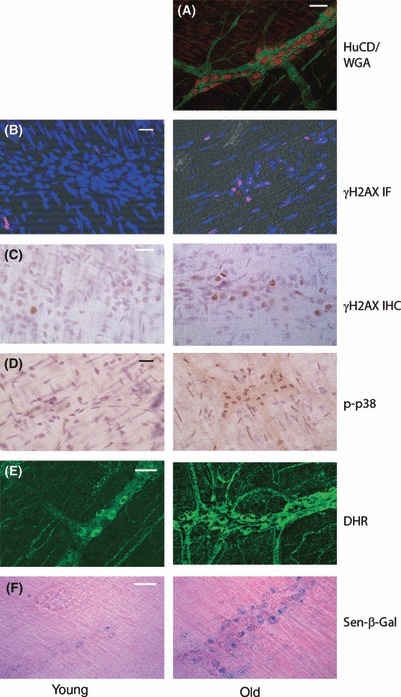
Myenteric ganglia in old mice are positive for multiple markers of the senescent phenotype. Intestinal strip preparations from young (5 months) and old (22–24 months) mice were stained for the following markers: (A) HuC/D and wheat germ agglutinin (WGA) to discriminate between neurons (HuC/D, red) and glial cells (WGA, green) (done in old mice only). Parallel preparations were immunostained for γH2AX by immunofluorescence (B), blue: DAPI, red: γH2AX foci) and immunohistochemistry (C), or activated p38MAPK (D). (E) Fresh preparations were incubated with dihydrorhodamine 123 (DHR-123, a marker for cellular peroxide production) or were stained for sen-β-Gal activity (F). Representative images are shown. The size marker bars indicate 20 μm.

To find out whether the results obtained in the brain would also hold for peripheral neutrons, we performed a similar analysis in myenteric ganglia (Fig. 3). In this system, neurons and glial cells were identified by staining with HuC/D and wheat germ agglutinin (WGA), respectively (Fig. 3A). Immunofluorescence (Fig. 3B) and immunohistochemistry (Fig. 3C) revealed frequent γH2AX foci in the large neuronal nuclei in myenteric ganglia from 22 to 24 months old mice. Furthermore, these cells were positive for activated p38MAPK (Fig. 3D) and for sen-β-Gal (Fig. 3F). ROS production in ex vivo preparations of myenteric ganglia was directly measured by staining with dihydrorhodamine 123 (DHR, Fig. 3E) (Thrasivoulou *et al*., 2006). All these signals were weaker in myenteric ganglia from young mice (Fig. 3A–F).

**Fig. 4:**
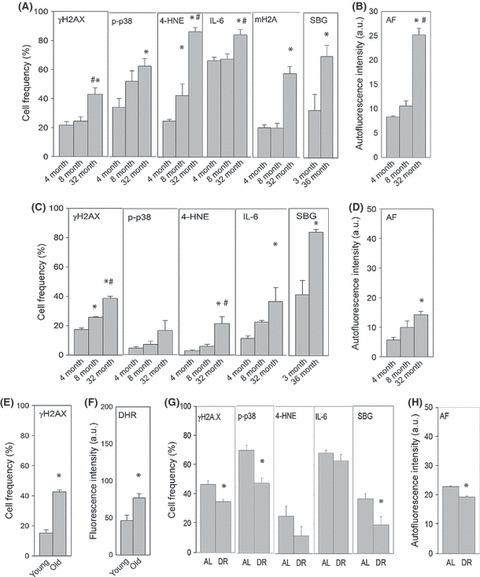
Aging increases the frequency of neurons with a senescence-like phenotype. (A) Frequencies of Purkinje neurons positive for the indicated marker (in %) at the indicated ages as measured by immunofluorescence and immunohistochemistry. (B) Purkinje neuron autofluorescence intensity (in arbitrary units) at the indicated ages. (C) Frequencies of cortical neurons positive for the indicated marker (in %). (D) Cortical neuron autofluorescence intensity (in arbitrary units). All data are mean ± SEM, from at least three animals per age group. * and # indicate significant differences to 4 and 8 months old animals, respectively (anova with post hoc Tukey, *P* < 0.05). (E) Frequencies of gut neurons positive for γH2A.X (in % of all neurons per ganglion) and (F) intensity of neuronal DHR-123 staining (arbitrary units) in young (5 months) and old (22–24 months) mice. Data are from > 15 ganglia from 2 or 3 mice per age group.*P < 0.01, *t*-test). (G) Frequencies of Purkinje neurons positive for the indicated marker (in %) and (H) autofluorescence intensity (in arbitrary units) in 17 months old ad libitum fed (AL) animals and in mice kept under dietary restriction (DR) from 14 to 17 months of age. Data are mean ± SEM, from three animals per age group. * indicate significant differences (*P* < 0.05, *t*-test).

For quantitative evaluation, images were scored blind by three independent observers. Results are given in Fig. 4. There were no major differences between markers that were assessed by both immunofluorescence and immunohistochemistry, and these data were combined. We used mice brains at ages of 4, 8 and 32 months to measure frequencies of γH2A.X-, phopho-p38MAPK-, 4-HNE, IL-6- and mH2A-positive neurons. Frequencies of Purkinje neurons positive for all these markers increased significantly with age (Fig. 4A) and so did the average autofluorescence intensity in Purkinje cells (Fig. 4B). Cryosections for the assessment of sen-β-Gal activity were available from mice brains at 3 and 36 months of age. There was a significant increase in sen-β-Gal-positive Purkinje neurons in the old group (Fig. 4A). All measured parameters (with the sole exception of activated p38MAPK, where the increase did not reach statistical significance) also increased in cortical neurons (Fig. 4C,D). A significant increase in oxidatively modified brain proteins with age was confirmed by probing whole brain lysates with an antibody against hyperoxidized peroxiredoxin (Fig. S6). Furthermore, frequencies of DDR-positive gut neurons increased with age (Fig. 4E) and so did the neuron-associated production of ROS (Fig. 4F).

Beneficial health effects of mild, late-onset, short-duration dietary restriction (DR) including reduced tumour incidence, improved fitness and cognitive function have repeatedly been described in mice and other mammals (Means *et al*., 1993; Spindler, 2005). We showed that a mild (28% reduction on average), short (3 months) DR started at 14 months of age was sufficient to reduce frequencies of senescent cells significantly in the transient amplifying zone of the intestinal crypt epithelium and in the liver, two organs that show significant increase in senescent cells during aging (Wang *et al*., 2010). Under the same experimental regimen, there was a clear tendency towards reduction for all measured markers of the senescent phenotype in Purkinje neurons. Short-term DR reduced significantly the frequencies of Purkinje cells positive for γH2A.X, phospho-p38 and sen-β-Gal (Fig. 4G) and resulted in lower autofluorescence intensities (Fig. 4H).

**Fig. 5:**
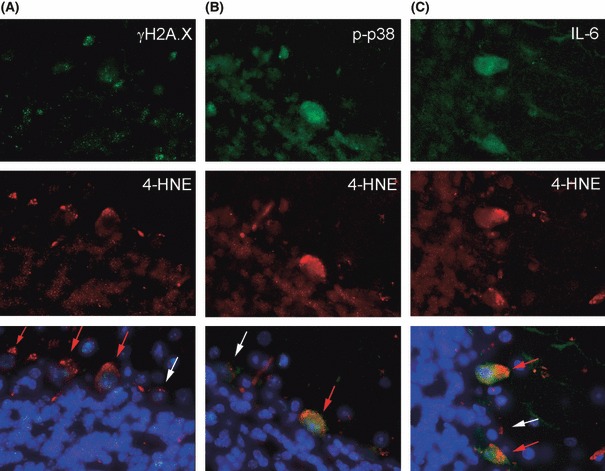
Purkinje cells are positive for multiple markers of the senescent phenotype as shown by double immunofluorescence for γH2A.X and 4-HNE (A), phospho-p38MAPK and 4-HNE (B) and Il-6 and 4-HNE (C) in cerebellar sections from 32 months old mice. Nuclei are stained with DAPI, which is shown on the merged images (bottom). Red arrows mark Purkinje neurons strongly positive for both antigens, white arrows indicate Purkinje cells that express both tested markers weakly or not at all. Bars indicate 20 μm.

Double immunofluorescence for various marker combinations showed that the same Purkinje neurons that were positive for γH2A.X were also positive for the peroxidation product 4-HNE, while adjacent γH2A.X-negative Purkinje cells showed reduced 4-HNE signals (Fig. 5A). Moreover, 4-HNE-positive, but not 4-HNE-negative, Purkinje cells also stained strongly for phospho-p38MAPK (Fig. 5B) and IL-6 (Fig. 5C). Immunohistochemistry of adjacent cerebellar sections confirmed that only Purkinje cells with activated DDR produced IL-6 (Fig. S7A). Similarly, most of the enteric neurons that produced high levels of ROS showed activated DDR as determined by immunohistochemical staining for γH2A.X straight after ex vivo measurement of ROS production in the same ganglia (Fig. S7B). Furthermore, the same neurons that produced high ROS levels were positive for sen-β-Gal activity (Fig. S7C). Together, these data suggested that DDR becomes more frequent with age in neurons and induces a senescence-like pro-inflammatory and pro-oxidant phenotype very similar to that in proliferation-competent cells.

**Fig. 6:**
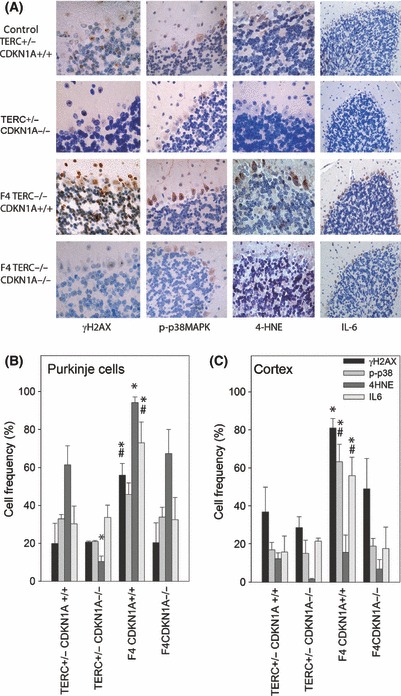
p21(CDKN1A) is necessary for the senescent phenotype in mouse brain neurons. (A) Cerebellar sections from mice with long telomeres (first and second row) either positive (TERC+/−CDKN1A+/+) or negative (TERC+/−CDKN1A−/−) for p21 expression and late-generation telomerase knockout mice with short telomeres (third and fourth row) positive for p21 expression (F4TERC−/−CDKN1A+/+, third row) or with additional p21 knockout (F4TERC−/−CDKN1A−/−, bottom row) were stained for γH2A.X, phopho-p38, 4-HNE and IL-6. Representative images are shown. Positive cells appear red/brown. (B) Quantitative evaluation for Purkinje cells. Data are percentage of positive cells, mean ± SEM. Numbers of mice per group were 3 (F4CDKN1A+/+), 4 (CONTROL and F4CDKN1A−/−) and 5 (TERC+/−CDKN1A−/−), respectively. * and # indicate significant differences to control and F4TERC−/−CDKN1A−/−, respectively (anova with post hoc Holm–Sidak test, *P* < 0.05). (C) Quantitative evaluation for cortical neurons. Symbols and statistics as in B).

To formally test this suggestion, we analysed brains from p21-knockout (CDKN1A−/−) mice. p21 is both a major inhibitor of cyclin-dependent kinases and an important transducer of the DDR signal towards p38MAPK that triggers the pro-oxidant and pro-inflammatory senescence-associated phenotypes in fibroblasts and other cells (Passos *et al*., 2010; Freund *et al*., 2011; Rovillain *et al*., 2011). In general, Purkinje cells in CDKN1A−/− mice of about 1 year of age showed weaker staining intensities for γH2A.X, phospho-p38MAPK, 4-HNE and IL-6 in direct comparison with parallel CDKN1A+/+ mice of the same age (Fig. 6A). However, counts of positive cells confirmed significant differences only for 4-HNE (Fig. 6B), most probably because of the relatively young age of these mice. Therefore, we aggravated DDR by telomere dysfunction. Mice heterozygous for the telomerase RNA gene TERC maintain their telomere length, while late-generation (F4) telomerase knockout (F4TERC−/−) mice develop widespread telomere dysfunction (Rudolph *et al*., 1999; Choudhury *et al*., 2007) resulting, among others, in serious memory deficits (Rolyan *et al*., 2011). Telomere dysfunction induced cell senescence in various tissues, specifically those with high proliferative activity like the transient amplifying zone of the crypt intestinal epithelium (Wang *et al*., 2009) and was associated with increased markers of oxidative damage in the same compartments (Passos *et al*., 2010). Brain neurons behaved similarly: Purkinje cells and cortical neurons showed increased DDR, p38MAPK activation, induction of oxidative damage and IL-6 production in F4TERC−/− mice with dysfunctional telomeres (Fig. 6). Importantly, all these changes are dependent on signalling through p21(CDKN1A) downstream of the DDR: levels of γH2A.X, phospho-p38MAPK, oxidative damage and IL-6 all returned to normal in Purkinje and cortical neurons from F4TERC−/−CDKN1A−/− mice (Fig. 6). In earlier work, an increase in DNA damage foci frequencies had been observed in a p21 knockout human fibroblast clone (Herbig *et al*., 2004). Reasons for this apparent difference to our data might include different cell culture conditions, clonality issues, strain and donor species differences or others. More recent data showed that p21 triggers a positive feed-forward signalling loop that induces increased ROS production in senescence, which in turn enhances DNA damage and frequencies of DNA damage foci. Thus, in proliferation-competent cells p21 acts both downstream and upstream of γH2A.X, and p21 reduction diminished DNA damage foci frequencies in human and mouse fibroblasts and tissues (Begus-Nahrmann *et al*., 2009; Passos *et al*., 2010). As shown in Fig. 6, this is also the case in mature neurons.

## Discussion

There is increasing evidence for cell senescence as a contributing, potentially causal factor for aging in mammals (Krishnamurthy *et al*., 2004; Herbig *et al*., 2006; Choudhury *et al*., 2007; Baker *et al*., 2008, 2011; Wang *et al*., 2009, 2010). This might be due to senescent cells acting as a continuous source of pro-inflammatory and pro-oxidant stimuli in tissues, inducing potent bystander effects and thus contributing causally to loss of organ homoeostasis and function in aging (Kuilman *et al*., 2008; Campisi, 2010; Nelson *et al*., 2012). However, despite long-established evidence for significant DNA damage levels in brain neurons and other postmitotic cells, the possibility of postmitotic cells developing a senescence-like phenotype had not been evaluated before.

Our present results show for the first time that mature postmitotic neurons develop a phenotype as a result of DDR signalling through p21(CDKN1A) that is indistinguishable from a typical senescent phenotype by analysis of a wide range of markers. These neurons are pro-oxidant and pro-inflammatory, show a marker for heterochromatinization and sen-β-Gal accumulation. This phenotype becomes more severe with aging. These data challenge the conventional view that senescence is exclusive to proliferating cells and that cell cycle arrest is the defining feature of the transition to a senescent state. They suggest that a potentially useful definition of senescence might be based on the signalling pathways causing the phenotypic changes downstream of a DDR, of which proliferation arrest might just be one among many.

It can be speculated that this neuronal senescence-like phenotype might be a relevant cause of age-related cognitive dysfunction. DNA damage response in the aging brain may be caused by telomere dysfunction, stress-induced DNA damage or any combination of both. In fact, very recent data suggest that telomeres might be sources of persistent DDR even without overt telomere shortening in various mice tissues including the brain (Fumagalli *et al*., 2012; Hewitt *et al*., 2012). The frequencies of neurons showing multiple markers of a senescent phenotype are very substantial, going well beyond 20% in Purkinje cells already in young mice brains. Purkinje neurons are among the most vulnerable neurons in the CNS, and significant losses of Purkinje cells with aging have been described in mice (Zhang *et al*., 2010). So far, the consequences of activated DNA damage signalling and an enhanced pro-inflammatory and pro-oxidant phenotype for function of the neurons involved are not known. Given these high frequencies, a complete loss of function in these neurons seems improbable. However, it is clear that cells with a senescent phenotype can profoundly affect other cells in their environment, ultimately inducing a senescent phenotype in surrounding normal cells with intact DNA damage check points (Nelson *et al*., 2012). Once initiated, a senescence-like phenotype in some neurons might thus become a major cause of its propagation from cell to cell. Pro-inflammatory cytokines and increased ROS production are well known to be major determinants of central nervous system dysfunction in aging and are strongly associated with the pathogenesis of age-related neuronal diseases (Schapira, 1998; Poon *et al*., 2004; Fernandez-Checa *et al*., 2010; Capuron & Miller, 2011). Our data suggest that aging neurons themselves, by developing a DDR-driven senescence-like phenotype, might become an important source of low-level, chronic pro-inflammatory and pro-oxidant signalling and thus a potential causal factor in brain and peripheral neuron aging.

## Experimental procedures

### Animals and tissue preparation

Male C57Bl/6 mice were used for the aging and DR studies as described (Wang *et al*., 2009, 2010; Cameron *et al*., 2011). F4TERC−/− and F4TERC−/−CDKN1A−/− mice were generated as described, maintained on a C57Bl/6J background (Choudhury *et al*., 2007) and used at around 1 year of age. All experiments were undertaken in compliance with UK Home office legislation under the Animals (Scientific Procedures) Act 1986. Animals were sacrificed by cervical dislocation, brains were immediately fixed in 2% buffered formaldehyde and paraffin embedded or immediately shock-frozen. Sections were cut at a thickness of 3 μm if not otherwise stated.

Small intestine strip preparations of the muscularis externa containing the myenteric plexus of neurons were dissected, and immunofluorescence staining and sen-β-Gal histochemistry was performed as described (Thrasivoulou *et al*., 2006; Wang *et al*., 2009). *In vitro* ROS levels in myenteric neurons were measured as described (Thrasivoulou *et al*., 2006) using dihydrorhodamine 123 (DHR-123) (10μM, Invitrogen), with an incubation time of 1 h at 37 °C. Two strip samples from each animal were prepared for DHR-123 staining; mean cytoplasmic fluorescence intensities of the neurons from four ganglia per sample were quantified (about 100 neurons per animal). This method has been described in detail and validated (Jallali *et al*., 2005; Thrasivoulou *et al*., 2006). To study the association between ROS production and DNA damage foci formation or activation of sen-β-Gal, the strip preparations were first subjected to the DHR-123 measurement as above. Fluorescence intensity of individual ganglia as well as gross morphology of the sample were recorded to enable identification of the same ganglia after fixation and immunohistochemical or histochemical staining.

### Immunohistochemistry, immunofluorescence, sen-β-Gal staining and autofluorescence

These were performed essentially as described (Wang *et al*., 2009, 2010; Lawless *et al*., 2010). Full experimental protocols are given as supplementary methods. The following antibodies were used (antibody validation studies given in brackets): γ-H2A.X (Ser139) mAb #9718 (Nelson *et al*., 2009) 1:250; 53BP1 #305 (Nelson *et al*., 2009) 1:1000 (Novus Biochemicals, Cambridge, UK); phosphor-p38 #4631 (Chlan-Fourney *et al*., 2011) 1:50; 4-HNE #HNEJ-2 (Imai *et al*., 2001) 1:50 (Japan Institute for the Control of Aging); Alexa Fluor 647-conjugated mH2A1 1:50 (gift from John Sedivy, (Kreiling *et al*., 2011); IL-6 pAb #6672 1:600 (Abcam, Cambridge, UK); Chicken anti-HuC/D #AB9346 1:100 (Chemicon, Oxfordshire, UK); Calbindin D28k #214 004 1:300 (Synaptic Systems, Goettingen, Germany); Rhodamine wheat germ agglutinin #RL-1022 1 μL mL^−1^ (Vector laboratories, Peterborough, UK); mouse IgG #I-2000 2 μg mL^−1^ (Vector laboratories); rabbit IgG #I-1000 2 μg mL^−1^ (Vector laboratories); Alexa Fluor 647 guinea pig #A21450, Alexa Fluor 594 mouse #A11005, Alexa Fluor 594 rabbit #A21207 all at 1:2000 (Molecular Probes, Paisley, UK); Fluorescein Avidin DCS #A-2011 1:500 (Vector laboratories); M.O.M Basic kit #BMK-2202 (Vector laboratories); Vectastain elite ABC kit (rabbit IgG) #PK-6101 (Vector laboratories).

Brain sections for immunofluorescence were incubated in 3% Sudan Black B (#199664-25G, Sigma, Gillingham, UK) in 70% Ethanol for 1–2 min to quench autofluorescence and washed three times in water before mounting with Vectashield mounting media (#H1400, Vector laboratories).

### Quantitative image evaluation

All conditions generating a single data set to be compared (i.e. aging, AL vs. DR, telomerase knockouts) were performed in a strictly standardized fashion in parallel, on the same day if possible. Microscopy was performed on a Nikon E800 equipped with Leica DFC 420 camera for immunohistochemistry or in widefield fluorescence using a Leica DM 5500B equipped with Leica DFC 360FX camera for immunofluorescence. Camera exposure and gain as well as lamp intensity were kept constant for each data set. Immunofluorescence images were captured using Leica LASAF v2.1.0 and immunohistochemistry images used LAS v.3.0.

Images were taken in a systematic random fashion, that is, without regard for the actual parameter under observation. Focal plane in fluorescent images was chosen based on maximum contrast in the DAPI image. All measurements were performed on stored original images.

For measurement of autofluorescence, 5-μm-thick nondeparaffinized, unstained brain sections were used. Average cytoplasmic fluorescence intensities were measured using equally sized circular regions of interest in ImageJ. Average background intensity was measured in the closest tissue-free region of the paraffin section and subtracted.

For all other parameters, cells were scored as positive or negative blindly and independently by three trained observers. For scoring, copies of the original images were used on which contrast and brightness were modified equally on all images of a set to generate an optimized compromise in feature recognition. This was also used to generate representative images for publication. For each parameter, criteria were established for positivity that were both operationable and sensitive. The following criteria were chosen:

γH2AX: at least one nuclear focus. Although various studies indicate that multiple DNA damage foci are associated with and probably necessary for cell senescence *in vitro* (Zou *et al*., 2004; Nelson *et al*., 2009; Passos *et al*., 2010), analysis of full image stacks would be necessary to quantify this on tissue sections (Hewitt *et al*., 2012). This was not deemed practical. Scoring cells with multiple foci resulted in a similar age-dependent increase but at lower absolute levels (data not shown).

mH2A: more than on nuclear focus. (Kreiling *et al*., 2011) used a single focus as criterion for positivity in mouse liver and lung. We found that most neurons showed a single focus already at young age but typically developed multiple foci with advancing age and therefore applied a more sensitive criterion.

4-HNE and sen-β-Gal: at least two perinuclear spots. Cytoplasmic markers are inferior in terms of cell identification and signal-to-background separation. Therefore, two spots close to the nucleus were required for positive identification.

Positivity for phospho-p38 or IL-6 was decided if the cellular signal intensity was clearly above background intensity seen in the isotype negative controls in agreement with standard histopathological usage.

Greater than 40 Purkinje cells and > 100 cortical neurons were counted per animal and stain and data are given as means ± SE of 3–6 animals/group.

### Western blotting

Levels of hyperoxidized peroxiredoxins were assessed by Western blotting using rabbit anti-Prx-SO3 antibody (Abcam, ab16830). Whole brains from three young (5 months) and three old (32 months) mice were homogenized in the presence of 100 mm N-Ethylmaleimide as described (Cox *et al*., 2010) to prevent artefactual Prx oxidation during sample preparation. Protein concentrations were determined by the Bradford method with bovine serum albumin as standard (Bio-Rad Laboratories, Hercules, CA, USA). Samples were analysed by 12% SDS–PAGE. Membranes were probed with an antibody against β-tubulin (Covance, Leeds, UK, MMS-422P) as loading control.

## Supporting information

**Fig S1** Further DDR markers in Purkinje neurons from old mice.

**Fig S2** Negative controls for immunofluorescence and immunohistochemistry.

**Fig S3** Purkinje neurons in old mice are positive for multiple markers of the senescent phenotype as shown by immunohistochemistry.

**Fig S4** Cortical neurons in old mice are positive for multiple markers of the senescent phenotype as shown by immunohistochemistry.

**Fig S5** Markers of a senescence-like phenotype in hippocampal neurons.

**Fig S6** Protein oxidation increases in old mice brains.

**Fig S7** The same cells are positive for multiple markers of the senescent phenotype.
