# Supplementary material for: Postmitotic neurons develop a p21-dependent senescence-like phenotype driven by a DNA damage response
Source: Aging Cell. 2012 Dec;11(6):996–1004. doi: 10.1111/j.1474-9726.2012.00870.x (PMC3533793; doi:10.1111/j.1474-9726.2012.00870.x)
Supplement: Supplementary file 1 [file acel0011-0996-SD1.doc]

**Supplementary Methods**

**1 H2A.X, p38 IHC, sen-β-gal staining after DHR on mouse gut neurons**

**1.1 Solutions for sen-β-gal:**

2mM Magnesium chloride

Potassium ferro-cyanide (Iron Bru): 5 mM Potassium ferrocyanide (Fwt 422.4), 5 mM Potassium ferricyanide (Fwt 329.2) in 100 ml water

Stain Base Solution (pH5.5): 150 mM sodium chloride, 40 mM citric acid, 12 mM sodium phosphate dibasic, make up the solution with autoclaved 2 mM Magnesium Chloride solution

X-gal: 1 mg/ml 5-bromo-4-chloro-3-inolyl-β-D-galactoside (X-gal) in dimethylformamide (20 mg/ml stock)

B-Gal Stain Solution (for 10ml): 8.8 ml staining buffer pH5.5, 1 ml Potassium ferro-cyanide (Iron Bru), 200 μ X-Gal

**1.2 Solutions for IHC:**

Primary Diluent: 22 mM Hepes buffer pH7.5, 0.1% DL-lysine, 5% Normal goat serum(NGS), 0.1% sodium azide, 0.1% triton, 1% BSA

Secondary Diluent at: PBS, 0.1% DL-lysine, 2% normal goat serum (NGS), 0.1% sodium azide, 1% BSA

Antigen retrieval solution: 2N HCl, pH 0.6-0.9**:**

**1.3 Procedure:**

1. Flush the gut and dissect into appropriate size
2. Stain tissue sections in DHR at 37°C for 45min
3. Take images of DHR staining on the confocal at x40 magnification
4. Transfer the tissue sections onto coated slides (4% APES)
5. Allow the tissue to air dry briefly
6. Fix the sections in 0.4% PFA/PBS – 1 min for sen-β-gal staining and 18 hours for IHC staining
7. Wash 3x 5 min in PBS

**1.3.1 For sen-β-gal staining**

1. After fixation incubate the sections in the β-gal staining solution at 37°C for 24h
2. Wash 2×3 min in PBS
3. Counterstain in nuclear fast red for 1 min
4. Wash in tap water until no more dye runs out
5. Mount with 50:50 glycerol:PBS

**1.3.2 For H2A.X and p38 IHC**

1. After fixation wash 3×5 min in PBS
2. 3×15 min in 22mM Hepes buffer pH7.5
3. Antigen retrieval with 2N HCl for 25 min at RT
4. Wash in 22mM Hepes solution for 2 min
5. Treatment in 3% H_2_O_2_ in PBS for 30 min
6. Rinse in 22 mM Hepes buffer
7. Permeablize in 0.5% triton, 0.5% BSA in PBS for 30 min
8. Block 1h in 22mM Hepes containing 5% NGS and 1% BSA
9. Incubate in primary antibodies H2A.X mAb rabbit (Cell signalling) at 1:250 or p38 at 1:50 overnight at 4°C. Dilute the antibodies in the primary diluents
10. Wash 3×15 min in 0.2% Tween PBS
11. Incubate in secondary antibody goat anti rabbit biotinylated for 30 min. Dilute 1:200 in secondary diluent
12. Wash 3×10 min in 0.2% Tween PBS
13. Apply AB complex for 30 min. 20µl reagent A + 1 ml PBS + 20µ reagent B (vector)
14. Wash 3×10 min in 0.2% Tween PBS
15. Apply NovaRed (vector) solution for up to 7 min
16. Wash 3 min in 0.2% Tween PBS
17. Counterstain in Hematoxylin for 5 sec
18. Wash in 0.2% Tween PBS for 1 min
19. 5 sec in 1% acid alcohol (1% HCl in ethanol)
20. Wash in 0.2% Tween PBS for 1 min
21. 20 sec in ammonia water
22. Wash in 0.2% Tween PBS for 1 min
23. Rinse in PBS
24. Mount with 50:50 Glycerol:PBS

**2 Immunohistochemistry staining on formalin-fixed paraffin sections**

**First day**

1. dewax and hydrate: 2×5min Histoclear, 2×5min 100% Ethanol, 5min 90% Ethanol, 5min 70% Ethanol, 2×5min water
2. antigen retrieval: microwave in 0.01M citrate buffer (high power for 3-4min until boiling and then 10 min medium power)
3. allow to cool down for 20-30min at room temperature

4 wash 2×5min in water

1. block endogenous peroxidise activity with 0.9% H_2_O_2_ in water for 30min
2. rinse twice in water
3. 5min in PBS
4. block non-specific binding with blocking solution for 30 min at RT normal goat

serum (NGS) 1:60 in 0.1%BSA/PBS

1. incubate in primary antibody overnight at 4°C, dilute antibody in blocking solution

**Second day**

1. wash sections 3×5min in PBS
2. incubate in biotinylated secondary antibody (goat anti rabbit) for 30min at RT

dilute 1:200 in blocking solution (PBS/BSA+NSG)

1. wash sections 3×5min in PBS
2. apply AB-Complex for 30min at RT(allow AB-C to stand for 30min)

10μl reagent A + 500μl PBS + 10μl reagent B

1. wash 3×5min in PBS
2. prepare Vector novaRed immediately before use
3. incubate sections at RT for 5-15min
4. wash 5min in PBS
5. rinse in water
6. counterstain in haematoxylin for up to 2min
7. wash 2×1min in water
8. wash in 1% acid alcohol for 5seconds
9. wash 1min in water
10. wash in ammonia water for 20seconds
11. wash 1min in water
12. dehydrate: 2×30sec in 95% Ethanol, 2×30sec in 100% Ethanol, 2×5min Histoclear
13. mount with DPX

**3 Immunofluorescent staining on formalin-fixed paraffin sections**

**First day**

1. dewax and hydrate: 2×5min Histoclear, 2×5min 100% Ethanol, 5min 90% Ethanol, 5min 70% Ethanol, 2×5min water
2. antigen retrieval: microwave in 0.01M citrate buffer (high power for 3-4min until boiling and then 10 min medium power)

3 allow to cool down for 20-30min at room temperature

4 wash 2×5min in water

1. 5min in PBS
2. block non-specific binding with blocking solution for 30 min at RT normal goat

serum (NGS) 1:60 in 0.1%BSA/PBS

1. incubate in primary antibody overnight at 4°C, dilute antibody in blocking solution (PBS/BSA+NSG)

**Second day**

1. wash sections 3×5min in PBS
2. incubate in biotinylated secondary antibody (goat anti rabbit) for 30min at RT

dilute 1:200 in blocking solution (PBS/BSA+NSG)

1. wash sections 3×5min in PBS
2. apply avidin DCS (1:500) in PBS for 20min at RT (vector lab)
3. wash 3×5min in PBS
4. incubate 5-10min in DAPI solution
5. wash 2×5min in PBS
6. incubate for 1-2min in Sudan black
7. rinse in water until water stays clear
8. dry briefly and mount in vectashield mounting media (vector lab)

**for double stain**

continue at point 13 with:

1. block non-specific binding with M.O.M. Mouse IgG blocking reagent for 1h at RT (90μl blocking reagent + 2.5ml TTBS)
2. wash sections 2×4min in PBS
3. incubate sections in M.O.M. diluents for 5min (600μl of protein concentrate +7.5ml of TTBS, extra volume later)
4. tip of the M.O.M. diluents and wipe of the excess
5. incubate with the primary mouse antibody overnight at 4°C

**Third day**

1. wash sections 3×5min in PBS
2. incubate in secondary antibody anti-mouse Alexa-594 (1:2000) in PBS for 30min at RT
3. wash 3×5min in PBS
4. incubate 5-10min in DAPI solution
5. wash 2×5min in PBS
6. incubate for 1-2min in Sudan black
7. rinse in water until water stays clear
8. dry briefly and mount in vectashield mounting media (vector lab)

**For triple stain**

continue at point 21 with:

1. block non-specific binding at RT with normal goat serum (NGS) 1:60 in 0.1%BSA/PBS
2. incubate with the primary guinea pig antibody in NGS/PBS/BSA overnight at 4°C

**Fourth Day**

1. wash sections 3×5min in PBS
2. incubate in secondary antibody anti guinea pig Alexa-647 (1:2000) in PBS for 30min at RT
3. wash 3×5min in PBS
4. incubate 5-10min in DAPI solution
5. wash 2×5min in PBS
6. incubate for 1-2min in Sudan black
7. rinse in water until water stays clear
8. dry briefly and mount in vectashield mounting media (vector lab)
