# Supplementary figures and images for: Postmitotic neurons develop a p21-dependent senescence-like phenotype driven by a DNA damage response
Source: Aging Cell. 2012 Dec;11(6):996–1004. doi: 10.1111/j.1474-9726.2012.00870.x (PMC3533793; doi:10.1111/j.1474-9726.2012.00870.x)

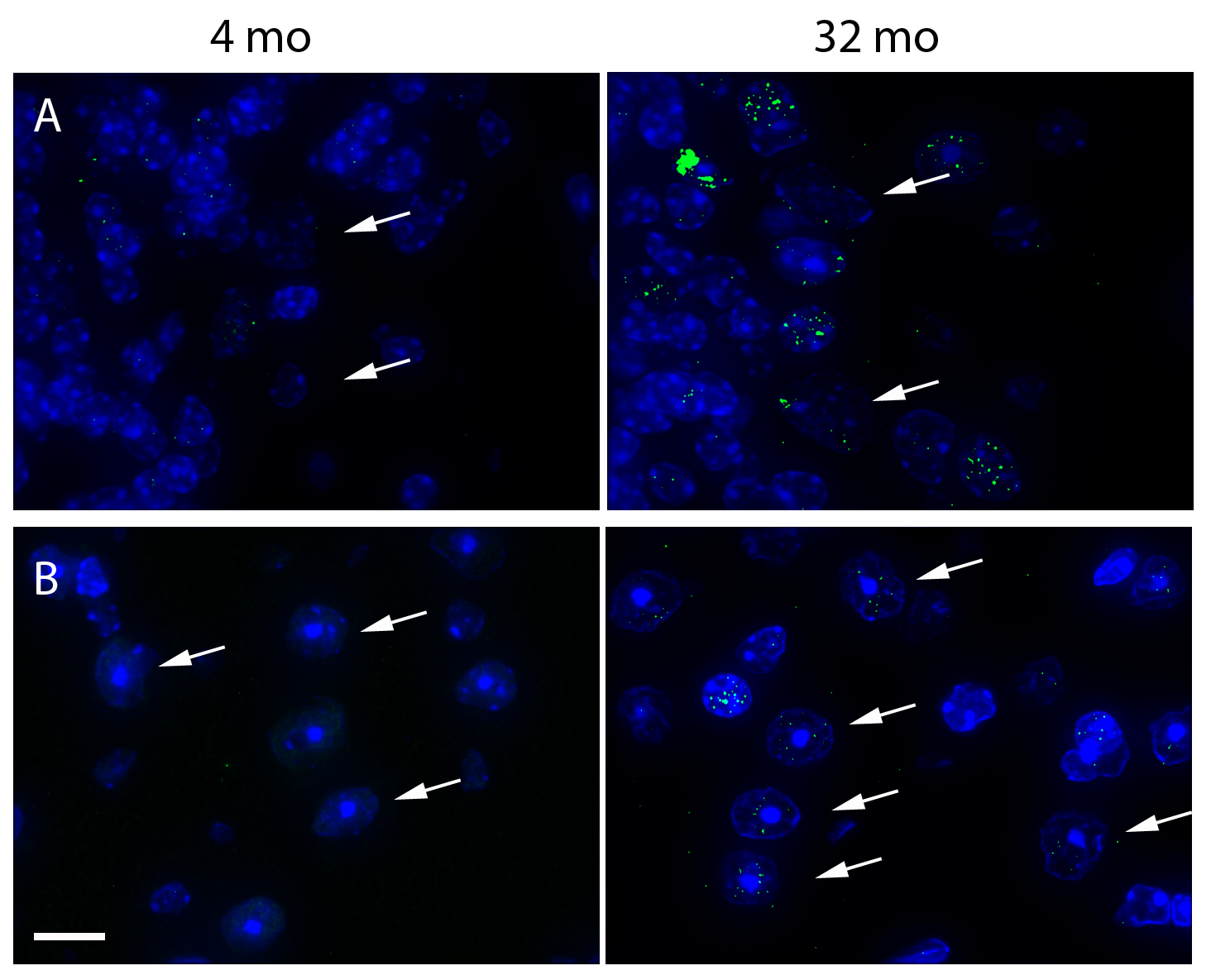

Supplement: Supplementary file 2 [file acel0011-0996-SD2.tif]

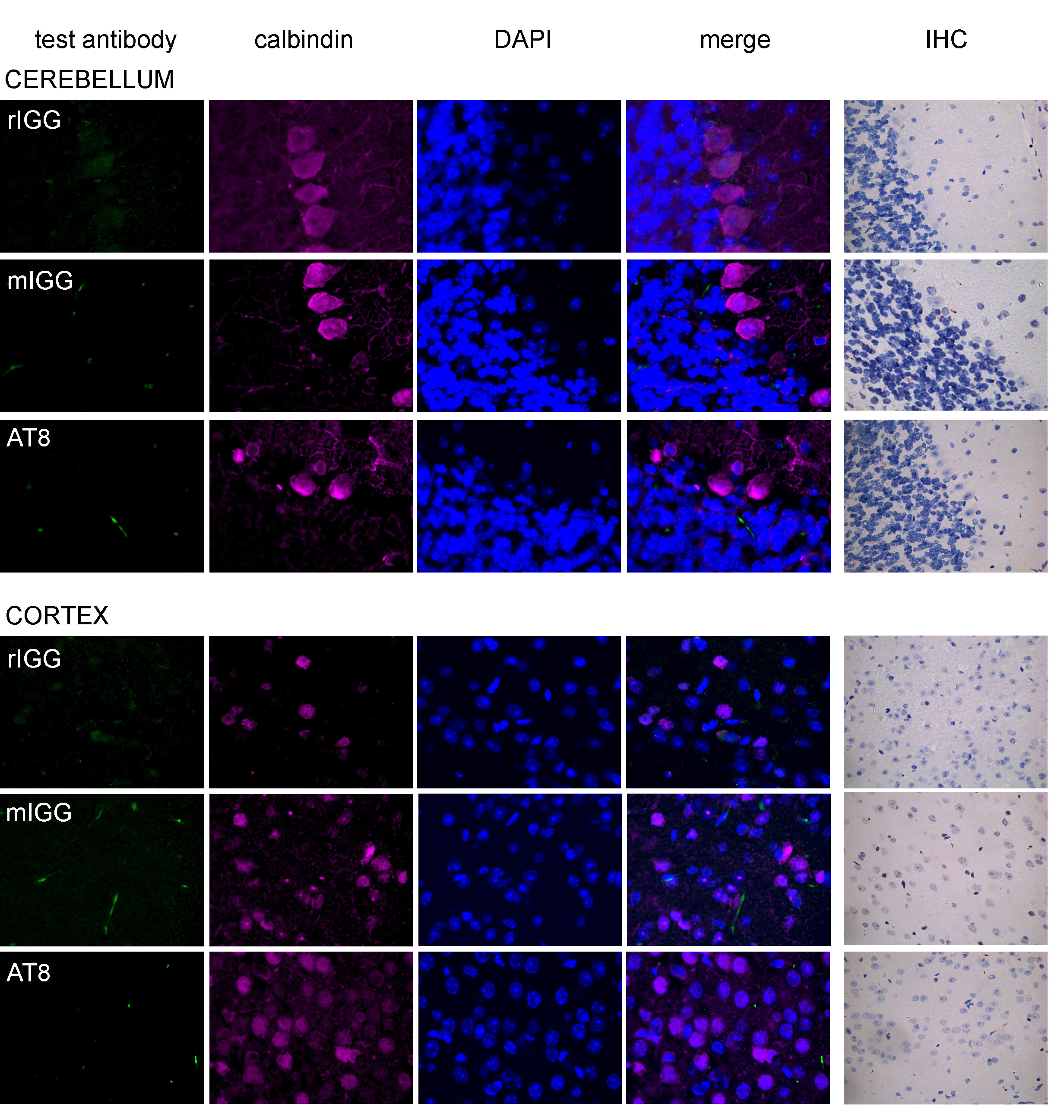

Supplement: Supplementary file 3 [file acel0011-0996-SD3.jpg]

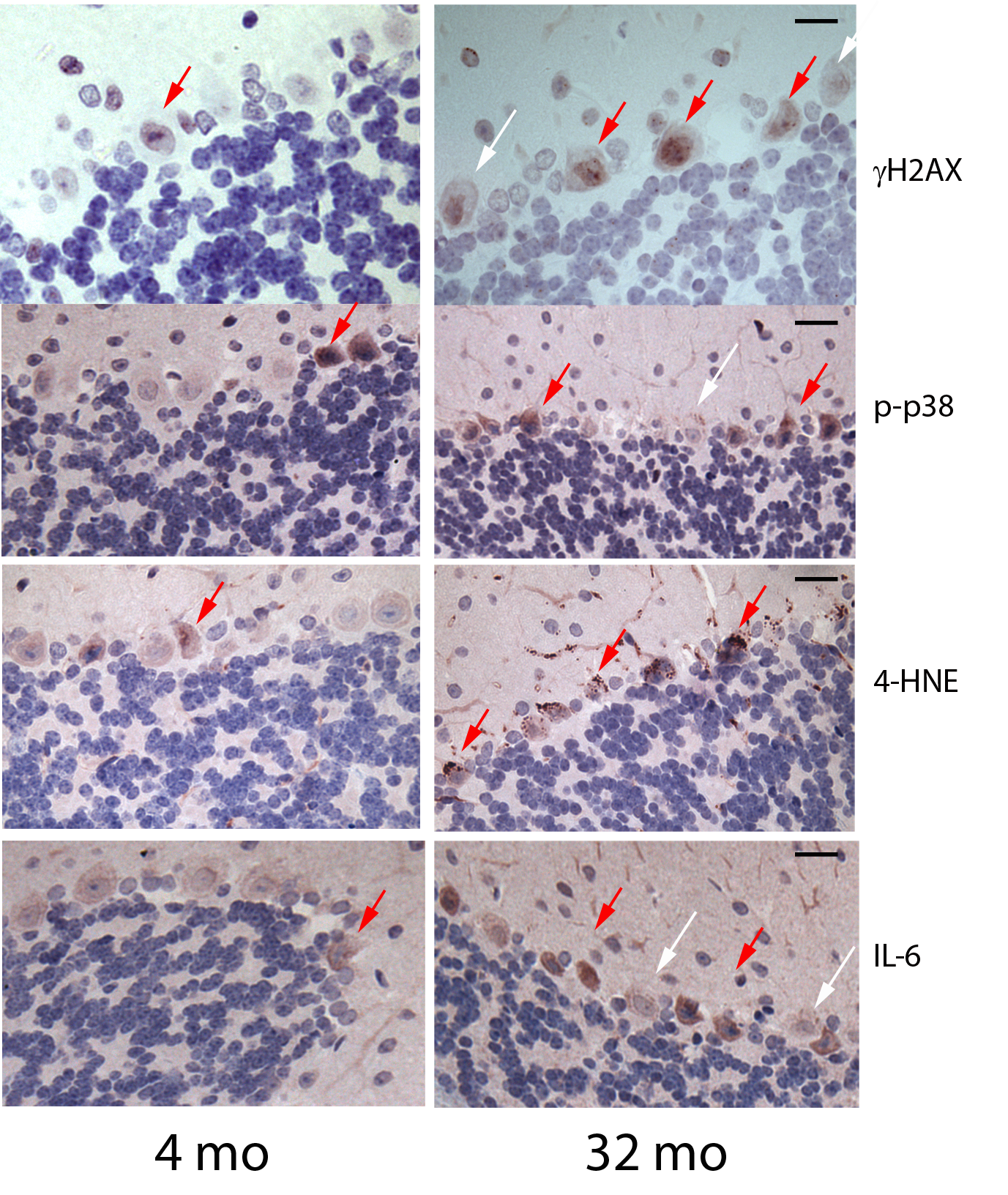

Supplement: Supplementary file 4 [file acel0011-0996-SD4.tif]

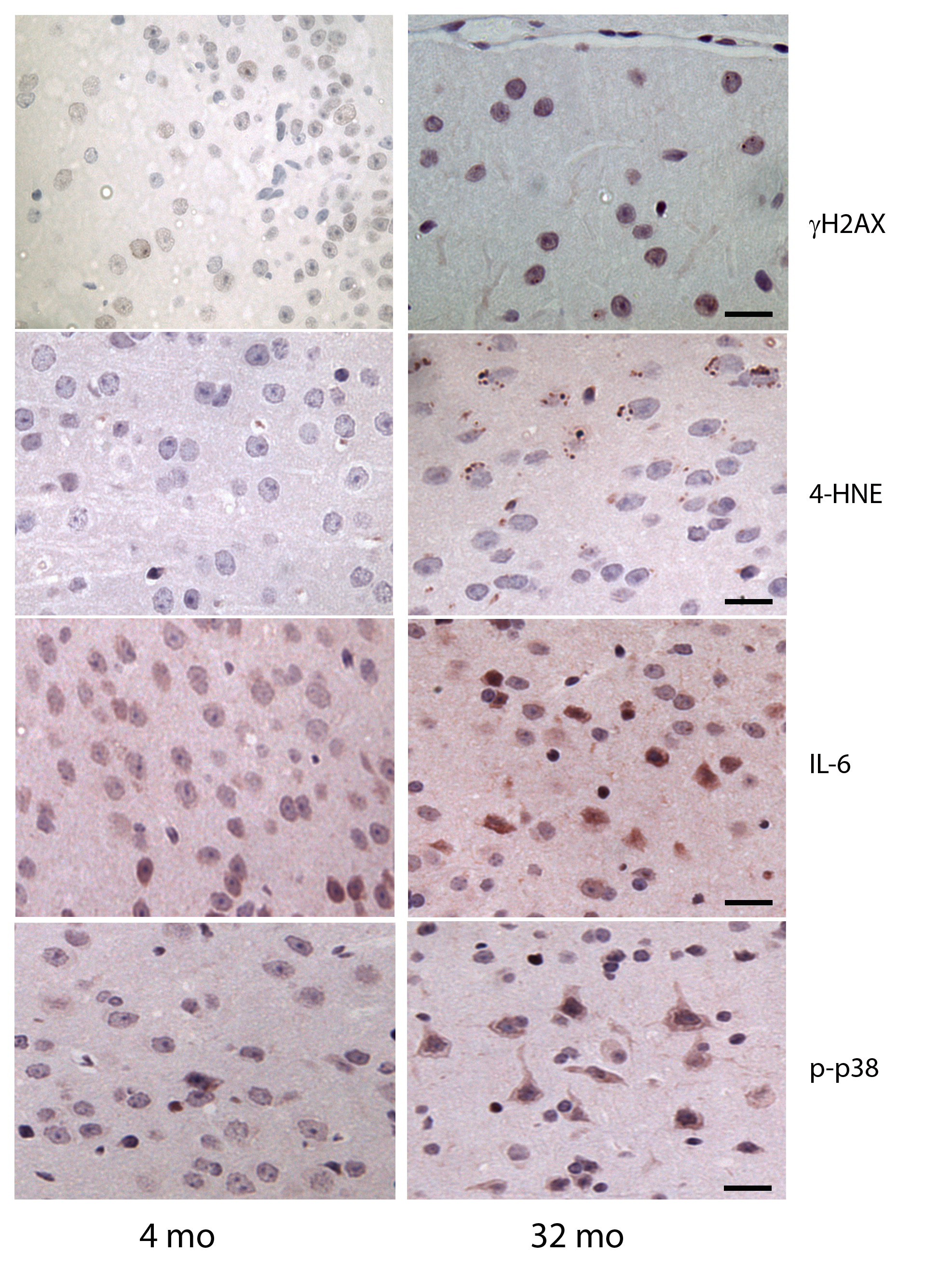

Supplement: Supplementary file 5 [file acel0011-0996-SD5.tif]

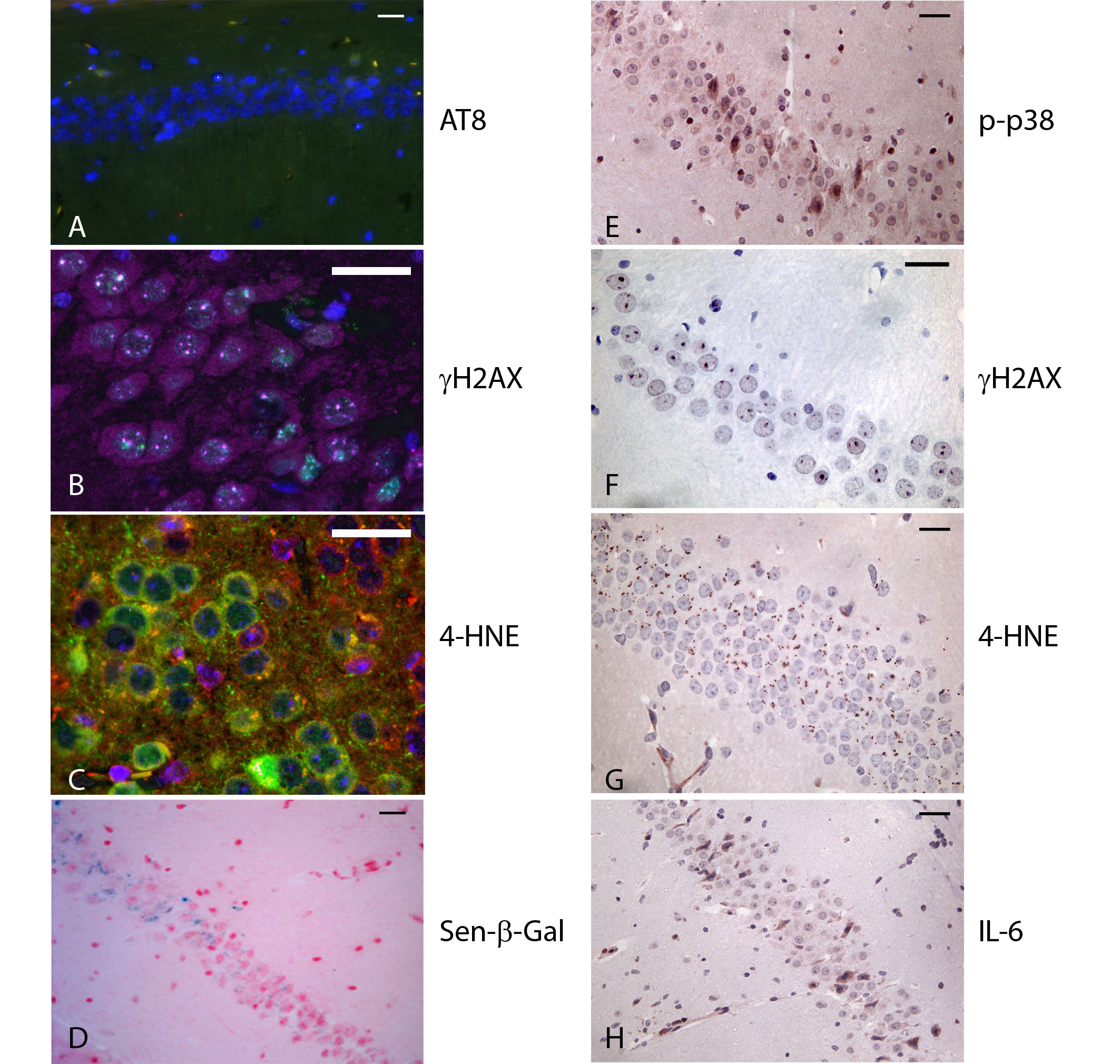

Supplement: Supplementary file 6 [file acel0011-0996-SD6.jpg]

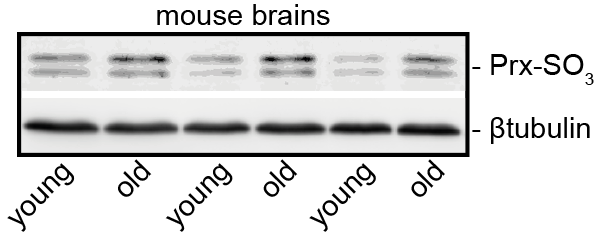

Supplement: Supplementary file 7 [file acel0011-0996-SD7.tif]

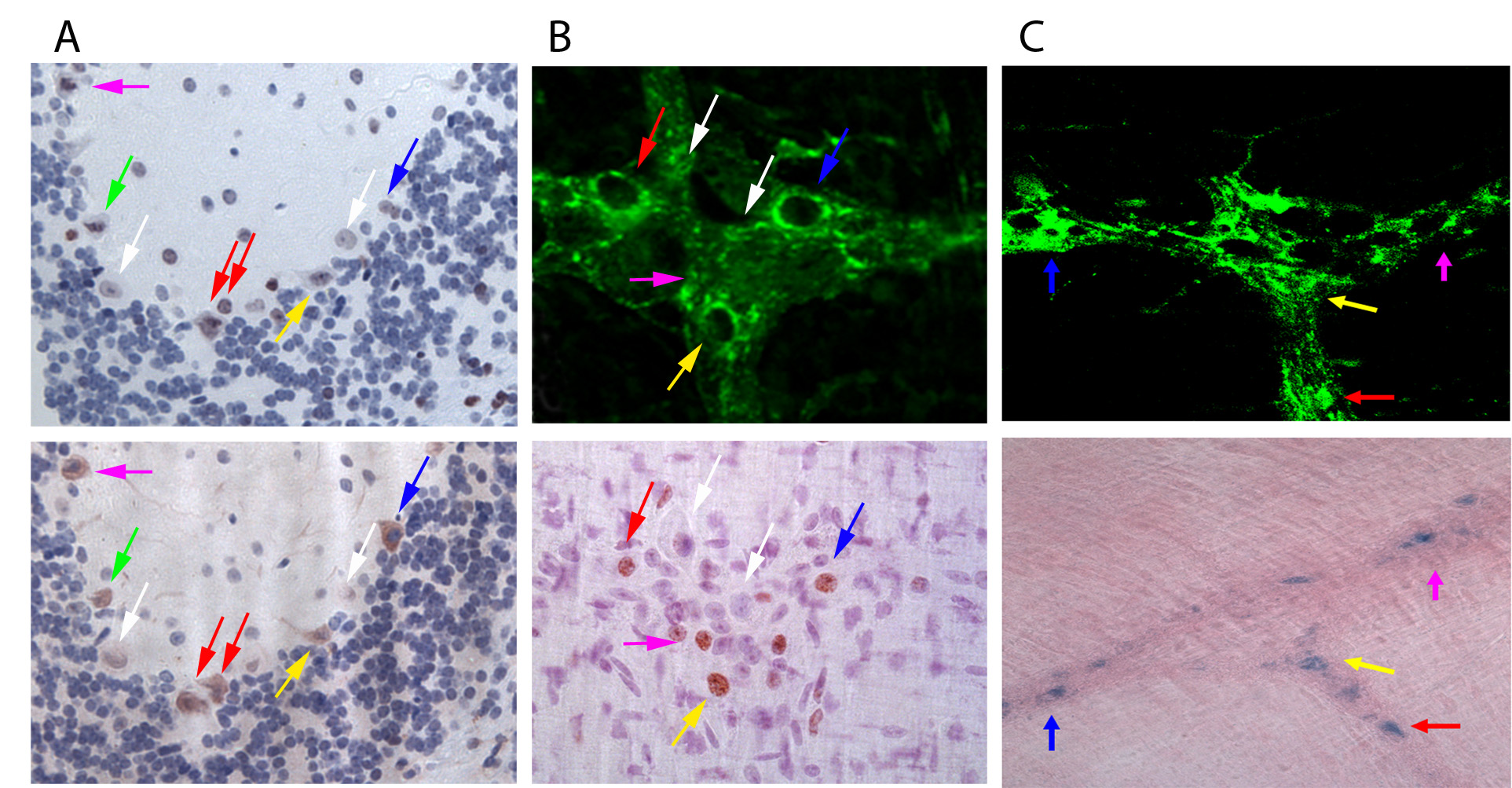

Supplement: Supplementary file 8 [file acel0011-0996-SD8.jpg]
