# Supplementary material for: Postmitotic neurons develop a p21-dependent senescence-like phenotype driven by a DNA damage response
Source: Aging Cell. 2012 Dec;11(6):996–1004. doi: 10.1111/j.1474-9726.2012.00870.x (PMC3533793; doi:10.1111/j.1474-9726.2012.00870.x)
Supplement: Supplementary file 9 [file acel0011-0996-SD9.doc]

**Supplementary Legends**

Fig. S1. Neurons in old (32 months) but not in young (4 months) mice are positive for 53BP1 foci. Cerebellar (A) and cortical (B) sections of mice brains at the indicated ages were stained with DAPI (blue, nuclei) and the antibody against the DNA damage foci component 53BP1 visualized by IgG-FITC (green). Image stacks were taken and deconvolved using Volocity 3D Image Analaysis Software (Perkin Elmer). Deconvolution settings were identical for all four images. Representative images are shown. Size marker bars indicate 20 μm. Arrows indicate probable Purkinje neurons (A) and cortical neurons (B), respectively.

Fig. S2. Negative controls for immunofluorescence and immunohistochemistry. Cerebellar (top) and cortical (bottom) sections from 32 months old mice were stained with rabbit (rIGG, first row) or mouse (mIGG, second row) isotype control antibodies or with an unrelated antibody (AT8, third row, all green). Sections were co-stained for the neuronal marker calbindin (purple). Nuclei were stained with DAPI. Immunohistochemistry was performed with the same negative control antibodies (right column). Representative images are shown from 3 examined animals.

Fig. S3.: Purkinje neurons in old, but not in young, mice are positive for multiple markers of the senescent phenotype as shown by immunohistochemistry for γH2A.X, activated p38MAPK, 4- -HNE and IL-6 (top to bottom). Negative controls are shown in supplementary Fig S2. Red arrows point to examples of Purkinje neurons positive for the marker in question, white arrows indicate negative neurons. Representative images of cerebellar sections from 4 (left) and 32 (right) months old mice are shown from 3 examined animals per age group. Bars represent 20 μm.

Fig. S4. Cortical neurons in old, but not in young, mice are positive for multiple markers of the senescent phenotype as shown by immunohistochemistry for γH2A.X, 4-HNE, IL-6 and activated p38MAPK (top to bottom). Representative images of cortical sections from 4 (left) and 32 (right) months old mice are shown from 3 examined animals per age group. Bars represent 20 μm.

Fig. S5. Markers of a senescence-like phenotype in hippocampal neurons. Representative images of hippocampal sections (CA2 – 3) are shown (3 animals, 32 months old). Bars represent 20 μm. A – C) Immunofluorescence. A) AT8 (green, negative control), DAPI (blue). B) γH2A.X (green), calbindin (purple), DAPI (blue). C) 4-HNE (purple), calbindin (green), DAPI (blue). D) Sen-β-Gal (blue), H&E (red). E) – H) Immunohistochemical stains for the indicated antibody visualized by peroxidase/NovaRed.

Fig. S6. Protein oxidation increases in old mice brains. Whole brain lysates from 3 young (4 months) and 3 old (32 months) mice were probed with an antibody recognizing hyper-oxidized peroxiredoxin (Prx-SO_3_). β-tubulin was used as loading control.

Fig. S7. The same cells are positive for multiple markers of the senescent phenotype. A) Immunohistochemistry for γH2A.X (top) and IL-6 (bottom) was performed on adjacent cerebellar sections from the same mouse brain (32 months old). Equally coloured arrows mark the same neurons on both sections, white arrows indicate negative cells. Purkinje cells that are positive for the DNA damage response marker γH2A.X also stain positive for IL-6. B) Sequential staining of the same myenteric ganglion was performed for DHR-123 (top), followed by immunohistochemistry for γH2A.X (bottom). Ganglia were imaged after the first staining and their position was marked on a graded slide. The second staining resulted in some distortion of ganglion structure as seen in the first image. The same neurons on sequential images are indicated by equally coloured arrows. Most γH2A.X-positive neurons show high levels of (cytoplasmic) DHR-123 fluorescence, while γH2A.X-negative neurons (white arrows) appear weaker in DHR-123. C) Sequential staining of the same myenteric ganglion was performed for DHR-123 (top), followed by staining for sen-β-Gal (bottom). Cells with high superoxide production are positive for sen-β-Gal. Intensities of the DHR-123 images in (B) and (C) are not directly comparable because these images were taken using different imaging settings from different animals.
